# Death from Effusion of Blood into the Left Lateral Ventricle

**Published:** 1848-01

**Authors:** 

**Affiliations:** Buffalo


ART. III.—*Death from Effusion of Blood into the left Lateral Ventricle. Report of the last illness, death, and Autopsy of the* Rev. ASA T. HOPKINS, *of Buffalo, aged about forty-two years.* By DR. HAMILTON.

For general languor of the body and loss of appetite, accompanied with a slight dimness of vision in one of his eyes, I had prescribed about the first of November, relaxation from his studies, out door exercise and mild tonics.

Sunday, the 19th of Nov., I was called to his room (until this time he had been out daily) and found him salivated from the use of about six grains of blue pill, which he had taken at his own suggestion. No other change had taken place in his symptoms. I prescribed mild diet, astringent washes, and a Seidlitz powder. He had himself suspended the tonics. Wednesday, as the swelling still continued, I washed his mouth, by means of a camel's hair pencil, with a strong solution of nitrate of silver; soon after which a hemorrage commenced from the mouth, and continued several hours, and until he had bled about half a pint, when I reached him, it had ceased spontaneously. He was directed to eat animal broths and moderate quantities of meat. On Friday (25th) he had a large evacuation of blood *per-ano.* Saturday morning, I found him in bed, but his face less swollen. He felt weak, and I advised him to continue the nutritious broths, animal food &c. Having occasion to leave town, I did not see him again until I was called in haste on Monday. On Sunday (27th) Dr. Bristol, in whose charge I left him, advised him to take moderately of the tonics—Port wine and bark—of which he took, during the day, three teaspoonfuls.

Monday forenoon, he spent in writing, and while sitting at his table, sealing a letter, he staggered, and fell from his chair. Ten minutes before, he had taken a teaspoonful of castor oil. I saw him within twenty minutes after the attack. He was completely paralyzed in his right arm and leg, and speechless. His eyes had a stupid, dull look, his lips flapped at each expiration, some froth gathered about his mouth, and his pulse was slow, but full. His feet were already in hot water. I bled him about thirty-two ounces, and applied mustard to various parts of his body, and cold to his head. The blood was highly buffed. After the bleeding he said "well," and "oh dear," three or four times. These were the last words he ever spoke.

In the evening, Drs. B. Burwell and Bristol, consulting physicians with myself, directed six leeches to the temple, cold continued to the head, sinapisms &c. The next day Dr. Josiah Trowbridge was added to the counsel. From the day of the attack until his death he received into his stomach free doses of salts and senna, Jalap and cream of tartar, with one drop of croton oil; also several cathartic injections were administered, and tolerably full evacuations were at length procured, cool applications were continued to his head, and blisters and sinapisms were applied to his neck and extremities during the last days, also nourishing broths and wine were given. His consciousness seemed but little impaired during most of the time, and he indicated his wishes by motions of his left hand. His sight and hearing gradually failed. The pulse varied from 90 to 120 at different periods, but was generally soft. But the action of his heart when examined by the ear was exceedingly violent. He seemed to suffer but little pain except when under the operation of cathartics. He died Saturday morning (Dec. 2d).

*Autopsy.*—Made on the Monday morning following his death. Present, Drs. Josiah and John S. Trowbridge, Bryant, and George N. Burwell, Bristol, White, and Barrett.

*Brain.*—Median meningeal artery congested. A deposite of lymph at two or three points about the top of the brain, on both hemispheres, lying within the tunica arachnoides. A clot of blood, measuring about an inch and a half in diameter, firm, and nearly round in form, occupying the left lateral ventricle, and much more in a fluid state occupying the horns of the ventricle. In all about three ounces. It had also saturated and softened, the structure of the brain in every direction immediately around the ventricle. The vessels through the whole structure of the brain were congested. Small amount of serum in the right ventricle.

No farther evidences of disease could be discovered within the cranium, or within the thorax or abdomen.

BUFFALO, Dec. 9., 1847.

